# Transcriptome-derived investigation of biosynthesis of quinolizidine alkaloids in narrow-leafed lupin (*Lupinus angustifolius* L.) highlights candidate genes linked to *iucundus* locus

**DOI:** 10.1038/s41598-018-37701-5

**Published:** 2019-02-19

**Authors:** Magdalena Kroc, Grzegorz Koczyk, Katarzyna A. Kamel, Katarzyna Czepiel, Olga Fedorowicz-Strońska, Paweł Krajewski, Joanna Kosińska, Jan Podkowiński, Paulina Wilczura, Wojciech Święcicki

**Affiliations:** 1 Department of Genomics, Institute of Plant Genetics, Polish Academy of Sciences, Strzeszyńska 34, 60–479 Poznań, Poland; 2 Department of Biometry and Bioinformatics, Institute of Plant Genetics, Polish Academy of Sciences, Strzeszyńska 34, 60–479 Poznań, Poland; 3 Department of Medical Genetics, Medical University of Warsaw, Pawińskiego 3c, 02–106 Warsaw, Poland; 4 Department of Molecular and Systems Biology, Institute of Bioorganic Chemistry, Polish Academy of Sciences, Piotrowo 2, 61–138 Poznań, Poland

## Abstract

Unravelling the biosynthetic pathway of quinolizidine alkaloids (QAs), regarded as antinutritional compounds of narrow-leafed lupin (NLL) seeds, is fundamental to best exploit NLL as food or feed. We investigated 12 candidate genes connected to QA biosynthesis, selecting them by transcriptomic and genomic approaches, from the landscape of genes differentially expressed in leaves of the high- and low-alkaloid NLL accessions. Linkage analysis enabled the assessment of the location of the candidate genes in relation to *iucundus*, a major locus of unknown identity, that confers reduced QA content in seeds. The key finding was the identification of APETALA2/ethylene response transcription factor, *RAP2-7*, cosegregating with the *iucundus* locus and located within a region with highly significant QTLs that affect QA composition. We additionally identified a 4-hydroxy-tetrahydrodipicolinate synthase (*DHDPS*) gene involved in L-lysine biosynthesis as being closely linked to *iucundus*. The distributed location of other remaining candidates (including previously known QA genes) across different linkage groups, also indirectly supports the transcription factor as a possible regulator of lupin alkaloid biosynthesis. Our findings provide crucial insight into QA biosynthesis in NLL. Additionally, we evaluated and selected appropriate reference genes for qRT-PCRs to analyse the expression levels of QA genes in NLL.

## Introduction

Quinolizidine alkaloids (QAs) are lysine-derived secondary metabolites that are distributed mainly in family Leguminosae and predominantly in genus *Lupinus*^1–3^. QAs protect plants against herbivores and pathogens, and because of their bitter taste and toxic effects on human and animals they are considered as antinutritional factors of food and feed^1^.

Previous studies showed that QAs were synthesized in aerial parts of plants and accumulated in maturing seeds with the highest synthesis levels at the flowering and fruit formation stages^1,3–5^. Results are still divided on whether QAs are exclusively transported or partially synthesised *in situ* in seeds^3^. The first step in QA biosynthesis is the decarboxylation of lysine by lysine decarboxylase (LDC)^6^ to give cadaverine amine. Cadaverine is then oxidized by amine oxidase (LaCAO)^7^, leading to a spontaneous intramolecular Schiff base formation, thus causing ring closure^4,5,8,9^. The addition of different functional groups and modifications gives the end products of QA synthesis, and their transport/storage forms^4,6–8,10–12^. QA ester formation is catalysed by an acyltransferase tigloyl-CoA:(−)-13α-hydroxymultiflorine/(+)-13α-hydroxylupanine O-tigloyltransferase (HMT/HLT; EC 2.3.1.93)^10,11^. An acyltransferase-like gene (*LaAT*) was also reported to be involved in the formation of QA esters or N-acylated polyamine conjugates^2^. To date, only a few studies have aimed to identify genes and characterize enzymes involved in QA biosynthesis in lupins^2,6,7,10,11,13–15^ and the regulatory mechanism is still to be explored.

Due to its great sampling depth, transcriptome sequencing (RNA-Seq) provides a nearly complete picture of transcriptional events, including rare transcripts, at a certain biological time point. Therefore, it has been widely used to characterize and annotate the transcriptomes of nonmodel plants, where the full gene repertoire is not known^16,17^. RNA-Seq technology has also been commonly employed in the comparative transcriptomic studies that elucidate the response of the plant to different treatment/conditions, and thus allowing the identification of candidate genes underlying particular traits as well as gene expression studies^16,18^. Lately, RNA-Seq has been used to identify candidate genes involved in various metabolic pathways and in the stress response in legumes (e.g., Rezaei, *et al*.^19^, Li, *et al*.^20^) and the biosynthesis of different alkaloid classes has been elucidated based on RNA-Seq data (e.g., Rai, *et al*.^21^, Cardenas, *et al*.^22^).

Recently, lupins have gained much attention as a valuable source of proteins (up to 44%), lipids (up to 14%), and fibre (up to 40% of seed mass), and could be an alternative to soybean in animal feed provided the antinutritional compounds in their seeds are reduced^23,24^. Consequently, many current lupin breeding programs aim to produce cultivars with strictly reduced alkaloid levels in their seeds. The accepted industry threshold for alkaloid levels is currently 0.02% of the seed dry weight^8^. In the Polish Lupinus Gene Bank (Poznan Plant Breeders Ltd., Poland), we previously found accessions of narrow-leafed lupin (*Lupinus angustifolius* L.) and white lupin (*Lupinus albus* L.) with much lower alkaloid content in their seeds (0.0005% and 0.016% of the seed dry weight, respectively)^24,25^. This demonstrates the improvement of breeding, and shows the high potential of collection as the source of low alkaloid material.

The major alkaloids (>1% of total alkaloids) detected in narrow-leafed lupin (NLL) are lupanine, 13-hydroxylupanine, angustifoline, and isolupanine^1,25^. Several single recessive genes related to reduced alkaloid content have been discovered in the seeds of plants with low-alkaloid NLL phenotypes^26,27^. Among them, the *iucundus* allele has been the most widely used in NLL breeding, although its identity is unknown^28^. A mapping population of low-alkaloid (sweet) 83A:476 and high-alkaloid (bitter) P27255 accessions (83 A:476 × P27255) was used to build high-density genetic maps that have provided markers tagging key agronomic traits and a background for synteny analysis with model legumes. The *iucundus* locus has been integrated into the first and all succeeding versions of the molecular genetic map^29–34^. This has provided an opportunity for the identification of markers linked to the alkaloid-content-related *iucundus* locus that may be suitable for marker-assisted selection (MAS) with the first attempt reported by Li, *et al*.^35^. Recently, a draft *L*. *angustifolius* cv. Tanjil transcriptome^32^ and a comprehensive draft NLL genome sequence^34^ have been reported (hereafter referred to as Tanjil transcriptome and draft NLL genome, respectively). Furthermore, genetic and genomic analyses of NLL were integrated using molecular cytogenetic tools, which provided insights into the cytomolecular organization and evolution of the NLL genome^36–40^.

The QA biosynthesis pathway is still obscure in lupins compared with other plants that produce alkaloids (e.g. steroidal glycoalakaloids in *Solanaceae* species^22^ and indole alkaloids in *Catharanthus roseus* L.^41^). Elucidation of the QA biosynthesis is fundamental to facilitate breeding efforts in providing valuable lupin sources that are used for animal feeding and human consumption. In this study, we sought to identify genes involved in QA biosynthesis by comparative transcriptome analysis of leaf tissue, derived from NLL accessions with contrasting seed alkaloid content. The availability of high resolution NLL linkage maps provided a valuable platform to identify the genetic positions of QA candidate genes as well as to detect quantitative trait loci (QTLs) associated with total QA content and relative abundance of individual QAs in seeds. Our research focused especially on those differentially expressed genes (DEGs) that were also cosegregating or closely linked to the major alkaloid *iucundus* locus and major QTLs underlying alkaloid composition. The results of this inquiry provide novel insight into the complex molecular mechanisms surrounding QA biosynthesis in NLL, and broaden our knowledge associated with the alkaloid metabolism in plants.

## Results

### RNA-Seq and P27255 transcriptome assembly, merging, and annotation

When leaf-derived cDNA libraries of two low-alkaloid (sweet) and two high-alkaloid (bitter) NLL accessions were sequenced (Table 1) and low quality and adaptor sequences were filtered, 632,431,153 high-quality reads remained (90.9% of processed reads). A total of 167,062,967 high-confidence short RNA-Seq reads for the reference P27255 transcriptome (pooled from two replications) were incorporated into the *de novo* assembly. The high-alkaloid accession P27255 was chosen to assemble the reference transcriptome, as we expected that genes involved in QA biosynthesis are upregulated in bitter accessions.

**Table 1:** Total quinolizidine alkaloid (QA) content and relative abundance of individual alkaloids of narrow-leafed lupin (NLL) accessions used for RNA-Seq and qRT-PCR analyses.

NLL accession	Catalogue No^†^	Total QA content (% of seed dry weight)	Relative abundance of individual alkaloids (%)				Applied in
			Angustifoline	Isolupanine	Lupanine	13-hydroxylupanine	
**high-alkaloid accessions**							
P27255 (wild landrace, Morocco)	—	2.72716	13.69	2.16	67.75	16.40	RNA-seq
Vitigudino-1 (wild landrace, Spain)	95706	2.59020	23.27	0.40	17.06	59.27	
BRGC-10275	95919	2.19975	18.47	0.26	33.02	56.76	qRT-PCR
Population B-549/79b	95742	2.37655	21.13	1.68	15.43	61.76	
Morsico Pop.1100	95946	2.43045	22.80	0.17	43.07	33.96	
Population-4	95878	2.49750	24.81	0.26	12.76	62.16	
Population-22746	95868	2.50240	24.90	0.25	9.82	65.03	
Population B-529/79	95732	2.51235	15.57	0.19	58.52	25.68	
Badajoz-3	95708	2.87505	19.64	0.29	13.63	66.45	
**low-alkaloid accessions**							
83A:476 (breeding line, Australia)	—	0.08290	11.65	1.65	65.36	21.34	RNA-seq
W-225 (breeding line, Poland)	—	0.00231	8.22	5.03	58.95	27.80	
Stadolishchienskij L-569	96128	0.00042	16.81	—	22.26	60.86	qRT-PCR
W-269	—	0.00095	4.06	5.75	46.31	43.89	
Geeburg	96208	0.00547	—	—	71.54	28.50	
Gunguru	96162	0.01274	9.95	2.00	50.23	37.83	
W-226B	96224	0.01459	1.49	2.56	69.64	26.31	
W-197	96222	0.02499	1.80	5.25	71.02	21.94	
Borweta	96194	0.02992	5.10	4.25	73.06	17.58	

^†^Polish Lupinus Gene Bank (Poznan Plant Breeders Ltd., Wiatrowo Branch, Poland).

**Figure 1:**
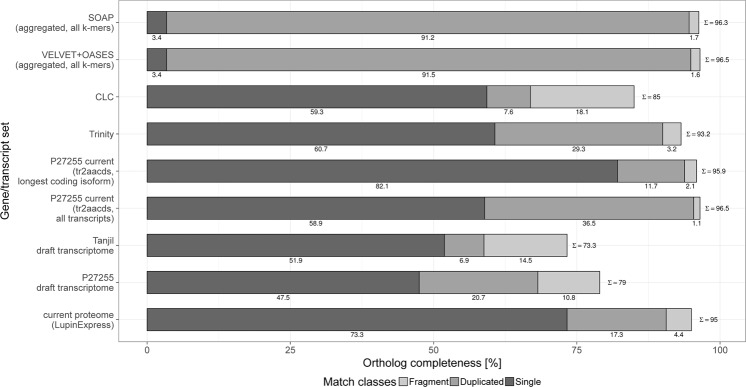
Comparison of individual and merged assemblies for P27255 accession based on single-copy ortholog completeness by BUSCO v3. The individual assemblies obtained using four methods (CLC Genomics Workbench, SOAPdenovo-TRANS, Trinity, VELVET + OASES) are shown. The entire merged tr2aacds transcriptome as well as the non-redundant version (using the predicted isoform with highest coding potential (longest coding sequence) for each of the predicted loci) are also shown. Our assessments of the previously available Tanjil and P27255 transcriptomes^32^ as well as recently available draft NLL proteome^34^ are included for comparison. Single indicates full-length orthologs; Fragment indicates fragmented orthologs; and Duplicated indicates duplicated orthologs. The numbers under the bars indicate the percentages of each ortholog and the summed total (∑) is also indicated.

After merging redundant transcripts from individual assemblies using the tr2aacds pipeline, we obtained 147,912 transcripts (average length 978.33 bp, N50 1750) and 65,835 candidate loci. The resultant merged sequence is hereafter referred to as the P27255 transcriptome. The average GC content of the P27255 transcriptome was 40.09%. Assessment of ortholog completeness (BUSCO v3 with the *Embryophyta*-specific set of single-copy orthologs) showed that the merged assembly captured more single-copy orthologs than any of the four separate assemblies and contained a low percentage of duplicated data (Fig. 1). A total of 28,769 candidate loci (43.7% of the total loci) were annotated using BLAST2GO, and gene ontology (GO) terms were assigned to 22,703 candidate loci (78.9% of the annotated loci).

### Differential gene expression, QA candidate gene selection, and genetic mapping

We detected 1489 differentially expressed transcripts (probability of equal expression <0.0001) in the bitter versus sweet accessions of NLL (2 accession in 2 replicates for both bitter and sweet); 1092 were significantly upregulated and 397 were downregulated in bitter accessions (posterior probability of differential expression ≥0.9999). We selected a subset of 962 transcripts with more than two-fold higher expression in bitter accessions (estimated posterior fold change (postFC) >2) for analysis (Supplementary Table S1) with further restriction to a set of 550 transcripts present across both bitter and sweet accessions.

To identify QA candidate genes, we focused mainly on the P27255 transcriptome. Moreover we used the draft NLL genome sequence^34^ that was released while we were analysing our results, to directly investigate the *iucundus* region in pseudochromosome NLL-07. A total of 12 candidate transcripts were selected; 11 chosen based on the functional annotation (differentially expressed representatives of transcription factor (TF) families and enzymes associated with legume secondary metabolism) and one that we identified as adjacent to the *iucundus* locus in the draft NLL genome (associated with primary metabolism) (Table 2). The candidate sequences were used in linkage analysis to determine their genetic positions in relation to the major alkaloid *iucundus* locus. The candidates were mapped to genome positions using the draft NLL genome (Table 2). Following analyses were focused on candidate QA genes closely linked to the *iucundus* locus.

**Table 2:** Selected alkaloid candidate genes: their differential expression (RSEM), genetic positions on the linkage map and genome positions on the draft NLL genome. PostFC, posterior fold change (bitter vs. sweet NLL accessions).

Category	Transcript name	Marker name	PostFC	Mean expression		Linkage group (NLL)	Pseudo-chromosome/Scaffold	Range	Locus name in NLL genome/NCBI GeneID	Blast2Go best hit	E-value
				High-alkaloid (bitter) accessions	Low-alkaloid (sweet) accessions						
genes involved in plant secondary metabolism	P27255_010872	CCR	449.31	659.53	1.16	NLL-02	NW017726398.1 (Scaffold_170_31)	1310-42407:-1	None/LOC109340430	CCR2_ARATH Cinnamoyl- reductase 2	1.80E-164
	P27255_012169	CES1L	191.12	18907.19	98.62	NLL-08	NW017724693.1 (Scaffold_95_70)	13279-15441:1	TanjilG_32669/LOC109339754	CXE1_ACTER Carboxylesterase 1	3.00E-108
	P27255_011759	DFR1	1281.93	669.87	0.21	NLL-03	NLL-03	4350135-4353301:1	TanjilG_08193/LOC109343177	DFRA_MALDO Flavanone 4-reductase	0
	P27255_010739	F3H	404.57	729.48	1.49	NLL-17	NLL-17	908504-911404:-1	TanjilG_04815/LOC109332349	FL3H_MALDO Flavanone-3-hydroxylase	0
	P27255_007723	HMT/HLT^†/‡^	1.10	3318.52	3017.52	NLL-04	NLL-04	11047022-11049076:1	TanjilG_22250/LOC109345839	HLTT_LUPAL 13-hydroxylupanine O-tigloyltransferase	0
	P27255_007730	LaAT^†^	2194.94	2061.47	0.63	NLL-16	NLL-16	18717081-18720909:-1	TanjilG_21586 /LOC109328823	SCT_ARATH Spermidine coumaroyl- acyltransferase	4.70E-109
	P27255_002184	LaCAO^†^	1109.81	21991.03	19.51	NLL-15	NLL-15	14027256-14034216:1	TanjilG_00530/LOC109328478	AMO1_ARTS1 Copper amine oxidase	4.80E-163
	P27255_008143	LDC^†^	472.81	51282.05	108.15	NLL-15	NLL-15	20620438-20621925:-1	TanjilG_09726/LOC109327937	DCOR_DATST Lysine/ Ornithine decarboxylase	4.80E-172
	P27255_009671	LDOX	143.29	1597.39	10.84	NLL-18	NLL-18	14128050-14145940:1	TanjilG_00883/LOC109332918	LDOX_MALDO Leucoanthocyanidin dioxygenase	0
transcription factors involved in plant secondary metabolism	P27255_010054	MYB	138.47	131.14	0.64	NLL-15	NLL-15	9035457-9037873:-1	TanjilG_10046/LOC109327542	MY106_ARATH Transcription factor MYB106	1.10E-60
	P27255_008724	RAP2-7	98.85	1210.64	11.94	NLL-07	NW017728885.1 (Scaffold 162_1)	324979-328373:-1	TanjilG_07628/LOC10934203	RAP27_ARATH Ethylene-responsive transcription factor RAP2-7	5.10E-53
							NLL-04	17076841-17081935:1	TanjilG_14185/LOC109346040		
gene involved in L-lysine biosynthesis pathway	P27255_011214	DHDPS	4.88	498.5	101.86	NLL-07	NA (pseudogene)	15272215-15275743:-1	TanjilG_17658/LOC109352463	DAPA_SOYBN 4-hydroxy-tetrahydrodipicolinate chloroplastic	0

^†^Known quinolizidine alkaloid biosynthesis genes that have already been described for lupins.

^‡^This gene had similar expression levels in both sweet and bitter NLL accessions (see also main text).

Segregation analysis in 93 recombinant inbred lines (RILs) generated new transcriptome-derived markers that were mapped to the NLL linkage groups (LGs) of the Hane, *et al*.^34^ map to produce an enriched linkage map (Supplementary Table S2). Among the 12 markers, 10 fitted the expected Mendelian segregation and two showed significant segregation distortions (P < 0.05) that favoured the wild-type parent (Supplementary Table S3). These two markers were located in the *iucundus* region of NLL-07. The total map length with new markers incorporated was increased by 9.4 cM to 2447.6 cM, but the average interval size remained unchanged (0.82 cM).

**Figure 2:**
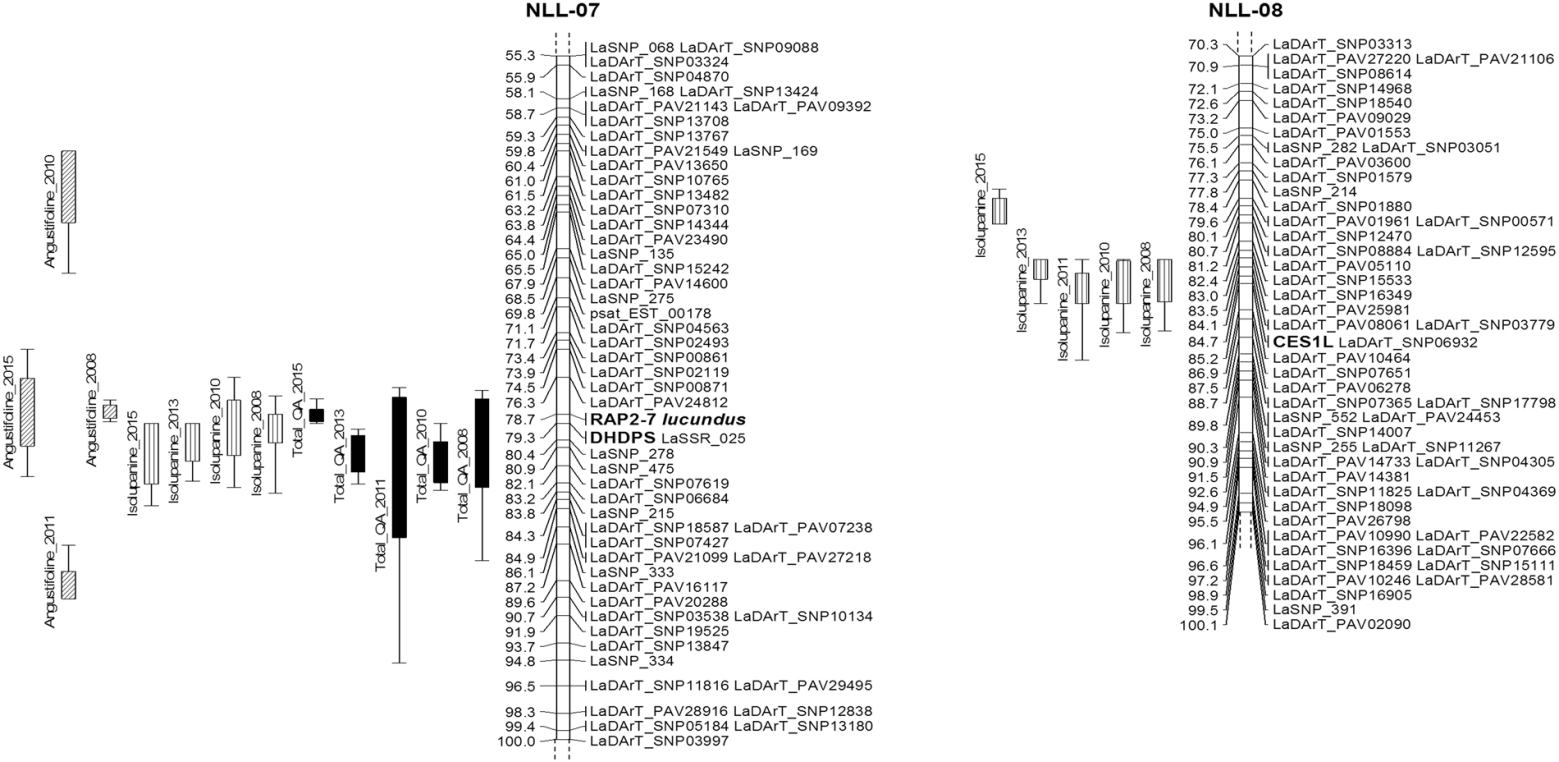
Reliable QTLs mapped to linkage groups NLL-07 and NLL-08 in the reference NLL genetic map^34^ enriched with new transcriptome-derived markers. For clarity, only a part of each linkage group is shown (NLL-07, 55–100 cM; NLL-08, 70–100 cM). Vertical bars on the left of each linkage group show the positions of the QTLs, with an inner block representing 1-LOD support interval and the end points determining 2-LOD support interval for each QTL location.

**Figure 3:**
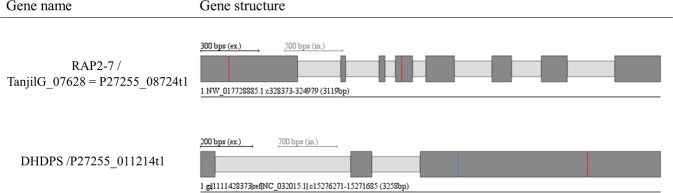
Gene models predicted by WebScipio^71^ (**a**) RAP2-7 based on sequence of longest coding isoform of candidate locus P27255_08724 (corresponding to TanjilG_07628), (**b**) DHDPS based on P27255_011214 candidate locus within the merged transcriptome. Dark grey bars and light grey bars mark exons and introns, respectively. Blue lines in the exon denote indel (compared to draft Tanjil genome), red lines denote mismatches (single nucleotide substitutions).

We found two transcriptome-derived markers that were closely linked to the *iucundus* locus and one of them, *RAP2-7*, represented by P27255_008724 candidate locus (postFC 98.8), co-segregated with *iucundus* (Supplementary Table S2, Fig. 2). *RAP2-7* is located in scaffold_162_1 that was not assigned to a pseudochromosome in the assembly v1.0. A BLASTN search of the P27255_008724 sequence against the annotated coding sequences set (cds v1.0) of the draft NLL genome^34^ revealed that its longest isoform corresponded to TanjilG_07628 (LOC109342033), which is annotated as APETALA2/ethylene response (AP2/ERF) TF (Table 2). Gene structure analysis revealed that TanjilG_07628 contained eight exons (Fig. 3). Investigation of differences between TanjilG_07628 and the longest coding isoform of P27255_08724 with WebScipio revealed two substitutions, M48V and S196R (the numbering is according to the XP_019435537.1 RefSeq protein for locus TanjilG_07628).

We confirmed the genetic position of *DHDPS* (P27255_011214 candidate locus with much lower postFC 4.88) by analysing the *iucundus* region in pseudochromosome NLL-07. *DHDPS*, which encodes chloroplast 4-hydroxy-tetrahydrodipicolinate synthase, was mapped 0.5 cM from the *iucundus* (Supplementary Table S2, Fig. 2). The *DHDPS* sequence is present in the current draft NLL genome, but the NCBI Gnomon pipeline reannotation marked TanjilG_17658 (LOC109352463) as a pseudogene. We detected a single mismatch and a single frameshift-causing deletion when the P27255_011214 transcript and TanjilG_017658 in the draft pseudochromosome NLL-07 sequence (NCBI:NC_032015.1) were compared using WebScipio (Fig. 3). We considered the frameshift-causing deletion of guanine may be one of the factors that distinguish sweet from bitter NLL.

Other transcriptome-derived markers mapped to LGs other than NLL-07 (Table 2 and Supplementary Table S2). Notably, some of the candidate genes identified in our analysis encode enzymes already known to be involved in QA biosynthesis, namely *LDC*^6^ (P27255_008143, NLL-15), *LaAT* ^2^ (P27255_007730, NLL-16), *HMT/HLT*^11^ (P27255_007723, NLL-04), and *LaCAO*^7^ (P27255_002184, NLL-15).

### Validation of RNA-Seq data by qRT-PCR

**Figure 4:**
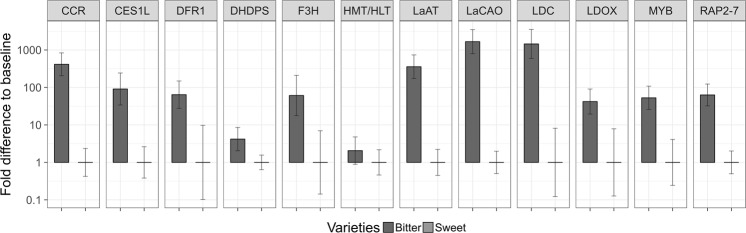
Expression profiles of 12 selected candidate QA genes by qRT-PCR. Bars indicate mean fold change expression in the bitter vs. sweet NLL accessions. Relative quantification was determined by qRT-PCR analyses normalized to three reference genes (alpha tubulin, ATP synthase, and alcohol dehydrogenase class-3).

To assess the accuracy of the RNA-Seq data, candidate QA transcripts were incorporated into qRT-PCR analysis. The eleven DEGs were found to be significantly differentially expressed (P < 0.0001) in the qRT-PCR analysis, whereas *HMT/HLT* was not (P = 0.003, 2.06-fold change). In the bitter accessions, *LaCAO* and *LDC* had the highest mean fold change (1666-fold and 1450-fold upregulation accordingly), followed by *CCR* (415-fold), *LaAT* (358-fold), *CES1L* (91-fold), *DFR1* (64-fold), *RAP2-7* (63-fold), *F3H* (61-fold), *MYB* (53-fold), *LDOX* (42-fold), and DHDPS (4-fold) (Fig. 4). The expression levels of QA candidate genes were normalized using the reference genes, alpha tubulin (*TUBA*), ATP synthase (*ATPsyn*), and alcohol dehydrogenase class-3 (*ADH3*) (see Supplementary Data S1 for details).

### QTL mapping and statistical analysis of bitter/sweet phenotypes

**Figure 5:**
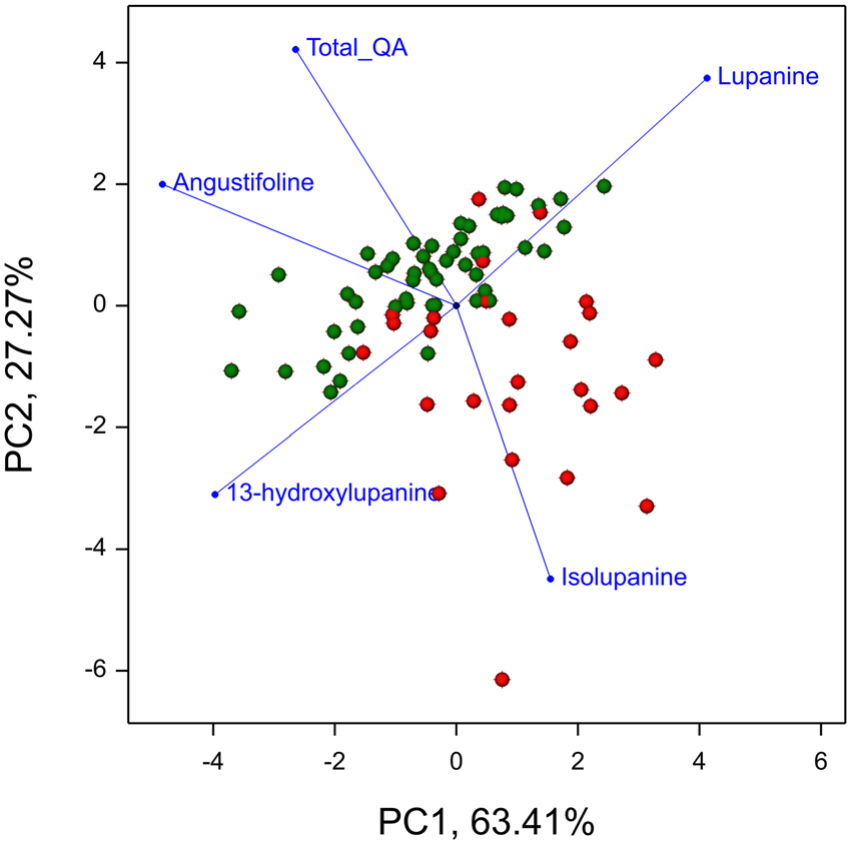
Principal component biplot constructed from mean values of total QA content and relative abundance values of individual QAs over five years for two subgroups of RILs. Red dots = *iucundus* allele (sweet), green dots = *Iucundus* allele (bitter).

The principal component biplot for two subgroups of RILs established based on their *iucundus* alleles shows the distribution of total QA content (estimated as % of seed dry weight) and relative abundance of individual QAs (% of total QAs) (Fig. 5). The low-alkaloid subgroup showed greater relative abundance of isolupanine, whereas the high-alkaloid subgroup had higher relative abundance of angustifoline, as well as the expected higher total QA content. The relative abundances of 13-hydroxylupanine and lupanine were similar in the two subgroups. Mean values for total QA content and relative abundance of individual QAs for the two RIL subgroups are given in Supplementary Table S4.

The distribution for total QA content was bimodal with apparent separation of low- and high-alkaloid RILs, whereas the distribution for relative abundance of isolupanine was left-skewed across the 5 years (Supplementary Fig. S1). The normality test showed that for some phenotypes the data did not follow a normal distribution (Supplementary Table S5). In the assessment of variance components, higher variance was observed for lines compared with years (Supplementary Table S6). The high broad-sense heritability justifies the conditions for QTL analysis (Supplementary Table S6).

QTL analysis performed to confirm the involvement of the *iucundus* region and detect other candidate genomic regions that may contribute to QA biosynthesis in NLL, identified 47 loci underlying the traits of total QA content and relative abundance of individual QAs across the 5 years (Supplementary Table S7). QTLs for the same and/or different traits were found to be colocalised on the LGs (overlapping map intervals) across years.

Colocalisation of reliable QTLs for different QAs was identified mainly in the *iucundus* region of NLL-07 (Fig. 2), confirming it as a major region involved in the QA biosynthesis pathway. The colocalising QTLs included those detected each year for total QA content, which individually accounted for 18.9–70.7% of the phenotypic variance (LOD values 7.8–27.8), as well as QTLs for relative abundance of angustifoline detected in four years (2008, 2010, 2011, and 2015) that were individually responsible for 12.6–56.4% of the phenotypic variance (LOD values 3.9–21.7). In both cases, QTLs originating from alleles of the paternal line (P27255) were responsible for increased total QA content and relative abundance of angustifoline in seeds. Colocalising QTLs for relative abundance of isolupanine detected in the *iucundus* region in four years (2008, 2010, 2013, and 2015) explained 11.4–20.3% of the trait variation (LOD values 3.6–6.9). These QTLs originated from alleles of the maternal line (83 A:476) and were responsible for increased abundance of isolupanine in seeds.

Colocalising QTLs for relative abundance of isolupanine across 5 years were also detected in the 79–90.2 cM map interval of NLL-08, which contains the carboxylesterase 1 (*CES1L*) gene that was mapped in this study (Fig. 2). These stable QTLs were individually responsible for 25.2–34.2% of the phenotypic variance (LOD values 7.2–9.9), originated from alleles of the paternal line P27255, and were responsible for increased abundance of isolupanine.

No consistent distribution of QTLs across years was detected for relative abundance of lupanine and 13-hydroxylupanine.

### Phylogeny of *RAP2-7* homologs in Viridiplantae genomes

A maximum-likelihood phylogenetic tree was constructed to reveal the evolutionary relationship between the candidate AP2/ERF TF gene *RAP2-7* (P27255_008724) and related genes in available Viridiplantae genomes (Supplementary Fig. S2). The NLL sequences clustered with other *AP2/ERF* sequences that were excluded in previous studies, namely At2g39250 (SCHNARCHZAPFEN, *SNZ*), At3g54990 (SCHAFLMÜTZE, *SMZ*), and At5g60120 (TARGET OF EARLY ACTIVATION TAGGED 2, *TOE2*)^42,43^. These genes were considered to encode a AP2/ERF domain, but were distinct from the typical ERF-type and more closely related to the AP2-type. A cluster of 65 AP2/ERF amino acid sequences, including three lupin sequences (TanjilG_07628 (LOC109342033), TanjilG_14185 (LOC109346040), and TanjilG_11298 (LOC109351048)), was formed in our phylogenetic tree. TanjilG_07628 was located in scaffold_162_1, TanjilG_14185 in NLL-04, and TanjilG_11298 in NLL-06 of the lupin genome. Differential expression analysis of these genes against sequences from the draft NLL genome^34^ showed that TanjilG_07628 and TanjilG_14185 were upregulated in the bitter accessions (postFC 65 and 500 respectively), whereas TanjilG_11298 was not significantly upregulated (postFC 1.8). Cross-referencing the draft NLL genome with the P27255 transcriptome revealed that TanjilG_07628 as well as several isoforms derived from TanjilG_14185 were putatively assembled as one candidate locus (*RAP2-7*, P27255_008724). However, when assessed by RSEM, the expression levels of the TanjilG_07628-derived isoforms were postFC 98.8, whereas TanjilG_11298 was not significantly differentially expressed. It is worth noting that, based on the consensus tree topology, TanjilG_07628 and TanjilG_14185 are recent paralogs (100% support), and are part of a strongly separated monophyletic subtree that includes AP2/ERF homologs from other legumes. Interestingly, the legume clade was identified as sister to another subtree that included the known Brassicaceae AP2/ERFs genes *SMZ* (*A*t3g54990, Bra007123) and *SNZ* (At2g39250). We identified *TOE2* as a distant homolog of TanjilG_07628. The three genes, *SMZ*, *SNZ* and *TOE2*, are known to repress flowering.

## Discussion

Based on the assumption that genes involved in QA biosynthesis are upregulated in leaves of bitter accessions, we assembled a reference transcriptome for high-alkaloid P27255, as the reference genotype very likely to express alkaloid-associated transcripts at consistent levels that would enable the assembly of good-quality, full-length transcripts. Recent works have shown that, typically, no single assembly captures all aspects of a transcriptome equally well, so an approach that combines multiple methods is more likely to produce a well supported assembly (e.g. Nakasugi, *et al*.^44^, Chen, *et al*.^45^). Thus, we used four individual assemblers that were merged in the EvidentialGene tr2aacds pipeline. The high-quality P27255 transcriptome that we obtained was more comprehensive than the previously reported Tanjil transcriptome^32^ and rivalled the currently available draft NLL genome^34^, regarding the ortholog completeness assessment (Fig. 1).

Based on the P27255 transcriptome and the *iucundus* region in the draft NLL genome^34^ we selected 12 candidate genes connected to QA biosynthesis and in the subsequent analyses we focused on the candidates most closely linked to *iucundus* locus and the major QTL underlying alkaloid composition. The linkage analysis of selected candidates confirmed that major QA genes colocalised with the major alkaloid *iucundus* locus on NLL-07. Mapping of QTLs underlying total QA content and relative abundance of individual QAs in NLL seeds confirmed *iucundus* as a major QA locus as well as identified other genomic regions involved in QA biosynthesis. The key finding of this study was the identification of *RAP2-7* (P27255_008724), an AP2/ERF TF that cosegregated with the *iucundus* locus, as well as *DHDPS* (P27255_011214, TanjilG_017658) that was located 0.5 cM from the *iucundus*. Both genes were located within an interval related to highly significant QTLs on NLL-07.

We demonstrated here, for the first time, that AP2/ERF TF is likely to be involved in the regulation of QA biosynthesis in NLL. The AP2/ERF TF superfamily has been divided into four families, AP2, ERF, RAV, and Soloist, based on the number of AP2 and other DNA-binding domains^46^, and has been well-studied in model plant systems, such as *A*. *thaliana* L. and rice (*Oryza sativa* L.). The ERF subfamily comprises 12 subgroups (I–XII) defined by Nakano, *et al*.^42^, and several members of ERF group IX were later found to regulate the biosynthesis of distinct classes of alkaloids in different plant species. For example, in *C*. *roseus* L., CrORCA2 and CrORCA3 regulated the synthesis of terpenoid indole alkaloids^47,48^; in *Nicotiana tabacum* L., the *NIC2* locus was involved in the synthesis of a pyridine alkaloid of nicotine^49^; in tomato and potato, GAME9 and JRE4 regulated the expression of steroidal glycoalakaloids^22,50^; and in *Ophiorrhiza pumila* Champ. ex Benth, OpERF2 regulated the production of monoterpenoid indole alkaloid camptothecin^43^. The phylogenetic relationships of NLL *RAP2-7* within the AP2/ERF TF superfamily (Supplementary Fig. S2) showed that it was a homolog of a distant subset of atypical *A*. *thaliana* AP2/ERF TFs, AT5G60120 (*TOE2*), AT3G54990 (*SMZ*), and AT2G39250 (*SNZ*), which were previously rejected from the Nakano, *et al*.^42^ and Udomsom, *et al*.^43^ large-scale phylogenies as outliers. Notably, *TOE2* and *SMZ* and its paralog *SNZ* belonged to an AP2/ERF clade that contained repressors of flowering and were predicted targets of microRNA miR172^51–53^. The downregulation of AP2-like target genes by miR172 resulted in the promotion of flowering^51^. The involvement of NLL *RAP2-7* in flowering regulation has not been reported so far and, to our knowledge, the association of alkaloid content and flowering time has not been investigated for lupins. Thus, despite the apparent analogy in the regulation of alkaloid biosynthesis in NLL and species with distinct alkaloid classes, such as *C*. *roseus*, *O*. *pumila*, and *Solanaceae*, a different molecular background may have evolved in lupins. This confirms the previous observation that unlike other groups of secondary metabolites, the biosynthesis and phylogeny of the numerous and structurally diversified classes of alkaloids are seemingly unrelated (or very distantly related)^12^.

We found that *RAP2-7* was located in an unplaced scaffold_162_1 that was characterized by long strings of unknown bases as well as transposon sequences, which may explain why it was not assigned to pseudochromosome NLL-07 in the currently available draft NLL genome^34^. Remarkably, the candidate *DHDPS* gene was identified in the *iucundus* region of pseudochromosome NLL-07. The structures of both scaffold_162_1 and the genomic region surrounding the *iucundus* locus imply some underlying assembly issues (the pseudochromosome region contains 48 gaps of unknown length, including a 77-kbp gap upstream of *DHDPS*), which could be indicative of the activity of repetitive elements during evolution. Significant segregation distortions for genetic markers neighbouring the *iucundus* locus have been reported by Boersma, *et al*.^33^, and were also apparent for the *RAP2-7* and *DHDPS* markers mapped in this study.

*DHDPS* homologs are typically involved in the L-lysine biosynthesis subpathway, a known precursor of QAs. The close location of *DHDPS* to *iucundus* and *RAP2-7* suggests but does not yet confirm the role of this NLL genomic region in QA biosynthesis. The other QA candidate genes investigated in our study were assigned to LGs other than NLL-07 (Table 2), and so were the known structural QA genes also identified in our analysis, namely *LDC* (NLL-15)^6^, *LaCAO* (NLL-15)^7^, *LaAT* (NLL-16)^2^, and *HMT/HLT* (NLL-04)^2^. Therefore, our results suggest that QA genes are not arranged in a cluster centered on the *iucundus* locus, or that the cluster is not very big. However, additional genes involved in QA biosynthesis and regulation may be recognized in the *iucundus* region when NLL genomic resources are enhanced. Indeed, clustering of nonhomologous biosynthetic genes of specific alkaloid pathways was reported previously in plants^54^. The GAME genes in the core steroidal glycoalkaloid biosynthesis pathway were found to be organized in metabolic gene clusters in the potato and tomato genomes^55^. Similarly, a cluster of genes encoding enzymes involved in noscapine biosynthesis was found in opium poppy^56^. Additionally, ERF-controlled gene clusters have been found in some plant genomes, including *N*. *tabacum* and other *Solanaceae* representatives^22,49^. The refinement of genetic maps, such as the very recently published high density map of Zhou, *et al*.^57^, may increase the identification of novel QA genes clustered in the *iucundus* region.

Despite the identification of several QA biosynthesis-related genes, the QA biosynthetic pathway in NLL is still far from being understood. Based on RNA-Seq data all the QA candidate genes were present in NLL genotypes with different alkaloid accumulation levels. Differential expression in the bitter vs. sweet accessions was also tested by qRT-PCR for twelve candidate QA genes (Fig. 4). Notably, in recent work of Yang *et al*.^7^ the researchers have identified and validated enzymatic function of a copper amine oxidase (*LaCAO*) found to be co-expressed with *LDC*. In their RNA-seq data and further qRT-PCR assay *LDC* and *LaCAO* expression was the highest in leaves, stems and pedicles. At the same time *LDC* expression was not detected in large seeds. The expression of characterised QA biosynthetic genes (*LDC*, *LaCAO* and *LaAT*) in bitter NLL accession P27255 and two sweet cultivars: Tanjil and Unicrop was also investigated by Frick *et al*.^15^. Significantly higher expression of these genes in leaf tissue points to leaves rather than seeds as the focal point of the alkaloid biosynthesis. Moreover, other, novel candidate QA genes were identified, which exhibited expression patterns similar to *LDC* and *LaCAO* in qRT-PCR analyses. These were chiefly annotated as major latex-like proteins (LaMPL1-like, LaMPL2-like and LaMPL4-like,) and were found to be located in NLL-10 (Lup019334) and NLL-06 (Lup015922 and Lup015923) groups in the draft genome^15^. The low level expression of QA candidates from this and other, recent studies^4,7^ in sweet accessions updates previously published results where *LDC* and *LaAT* expression was detected only in bitter NLL cultivars by gel-based RT-PCR^2,6^. Thus, the initial step of QA biosynthesis and some subsequent steps were not completely blocked at the transcriptional level in sweet NLL genotypes, as initially proposed by Hirai, *et al*.^14^. Furthermore, despite the different quantities, the same end products of QA biosynthesis were identified in both NLL genotypes. The observed downregulation of *LDC* in sweet NLL genotypes implied that the QA biosynthesis pathway was regulated at the initial step of lysine decarboxylation, whereas qualitative differences in QA composition of sweet and bitter RILs (Table 1) implied additional regulatory steps in the QA biosynthesis pathway downstream of lupanine. Our *HMT/HLT* gene expression results are in concurrence with previous studies that reported HMT/HLTase activity was similar in both sweet and bitter forms of *L*. *angustifolius*, *L*. *albus*, and *L*. *luteus*, and support the earlier hypothesis that *HMT/HLT* was regulated independently from genes that determine alkaloid accumulation and its expression was uncorrelated with final concentrations of alkaloids in seeds^11,13,14^.

As a whole, the growing body of evidence implies interaction of genes underlying QA biosynthesis in a form of gene network. Gene network reconstruction is a powerful approach for understanding the complex biological systems that underlie phenotypes^58^. The abundance of genomics/transcriptomics data based on next-generation sequencing technology enabled recently deeper investigations of gene networks in many plants, including legume representatives^59–61^. The insights from such datasets (or their lupin counterparts, once these become available), can be expected to allow for future quantitative as well as qualitative description of regulatory principles guiding secondary metabolism, including alkaloid biosynthesis.

Within the present study, we identified, for the first time, individual QTLs underlying the total QA content and relative abundance of major alkaloids in NLL seeds (Supplementary Table S7). Credible QTLs for total QA content as well as relative abundance of angustifoline and isolupanine were consistently detected over a period of 4–5 years, indicating that they were not affected by the environment. We uncovered strong support for the colocalisation of these QTLs in the *iucundus* region using the transcriptome-derived candidate *RAP2-7* and *DHDPS* loci (Fig. 2). Colocalisation of QTLs for different QAs may indicate that a gene in the colocalisation site determines the key step in the biosynthesis/accumulation of all compounds, possibly through a common regulatory mechanism. Stable QTLs in the *iucundus* region that accounted for the high phenotypic variability (Supplementary Table S7), confirmed that this region may govern the most decisive steps in QA accumulation. *RAP2-7* is likely the gene underlying the major QTL and it may be useful for MAS in breeding programs to lower QA levels in NLL seeds. The major QTL in NLL-08 for relative abundance of isolupanine contained the mapped QA marker *CES1L*, and its homologs are known to be involved in secondary metabolism in plants, including noscapine biosynthesis in the opium poppy^62^. No stable QTLs for relative abundance of lupanine and 13-hydroxylupanine were detected in the NLL genome. Similar tendencies were observed for the bitter and sweet RIL subgroups (Fig. 5).

In conclusion, our findings provide crucial insights into the QA biosynthesis pathway and its transcriptional regulation in NLL. The results strongly suggest that QA biosynthesis in this species is controlled mostly at the level of transcriptional regulation. We suggest that our characterization of *RAP2-7* is a worthwhile starting point for better disentangling the signalling and transcriptional regulatory networks associated with QA biosynthesis in this important legume crop. Moreover, the identified and characterized DEGs are a valuable resource to further explore the mechanisms underlying QA biosynthesis, for functional studies, and for the development of markers that are useful in MAS.

## Materials and Methods

### Plant material

Seeds of the investigated NLL accessions deposited in the Polish Lupinus Gene Bank and breeding programs were provided by Poznan Plant Breeders Ltd., Wiatrowo Branch (Poland). Seeds of the parental lines (83A:476 and P27255) and 93 RILs were provided by the Department of Agriculture and Food, Western Australia (DAFWA, Australia). All seeds were grown in early spring (March/April) with natural vernalisation in a field experiment in Wiatrowo, Poland. Mechanical weed control treatment as well as P_2_O_5_ (60 kg/h) and K_2_O (90 kg/h) fertilisers were applied.

Accessions used for RNA-Seq and qRT-PCR were grown in 2014 (Table 1). Young leaves were collected at flowering time and immediately frozen at −80 °C. Flowering time varied among lines and the sample collection proceeded for each plant when most of the flowers on the main stem inflorescence were open (erect standard petal).

For QTL mapping, the field trials included the RILs of the mapping population and parental lines, which were sown in a completely randomized design with two replicates (5 seeds per plot) in five growing seasons: 2008, 2010, 2011, 2013, and 2015. Seed samples for gas chromatography analyses were harvested from the RILs each year after full maturity.

### Assessment of total QA content and relative abundances of QAs in NLL seeds

Quantitative and qualitative QA composition were evaluated by gas chromatography (GC-2014; Shimadzu, Kyoto, Japan) to validate the alkaloid profiles of seeds from the selected accessions (Table 1) and from 80 representative RILs (mixture of seeds from a plot × 2 replicates, each growing seasons). Alkaloid extraction and analyses were conducted as described by Kamel, *et al*.^25^. The results were received as mean values from the two gas chromatography replicates for each RIL. Total QA values are the percentage of the sum of the major QAs (lupanine, 13-hydroxylupanine, angustifoline, and isolupanine) of the seed dry weight (% of seed dry weight). Relative abundance of major QAs were assessed as the percentage of total QAs (sum of all QAs = 100%).

## RNA and DNA extraction

Total RNA was isolated from 30 mg of ground leaf tissue using a SV Total RNA Isolation System Kit (Promega, Madison, WI). Total RNA concentration and quality were determined using a 2100 Bioanalyzer (Agilent Technologies, Palo Alto, CA) with a minimum RNA integrity number of 8. The extracted RNA was used for RNA-Seq and qRT-PCR analyses.

Genomic DNA was isolated from frozen, young leaves using a DNeasy Plant Mini Kit (Qiagen, Germantown, MD). DNA concentration and quality were determined using a NanoDrop ND-1000 (Thermo Fisher Scientific, Waltham, MA).

### cDNA library preparation and transcriptome sequencing (RNA-Seq)

Two low-alkaloid (sweet, 83A:476 and W-225) and two high-alkaloid (bitter, P27255 and Vitigudino-1) NLL accessions were used for RNA-Seq in two biological replicates (Table 1). cDNA library preparation was conducted in the Institute of Bioorganic Chemistry, Polish Academy of Sciences using a TruSeq RNA Sample Preparation Kit v2 (Illumina, San Diego, CA). Library sequencing was performed on an Illumina platform (HiSeq 1500, PE 2 × 75 bp, HiSeq v4 reagent sets) in the Medical University of Warsaw, Poland. The RNA-Seq data have been submitted to GenBank as short-read archives under the following accessions: SRR5723679, SRR5723680, SRR5723681, and SRR5723682 in BioProject PRJNA389154.

### Transcriptome *de novo* assemblies of accession P27255, merging, and annotation

The raw data were filtered to remove low-quality reads (<Q30) and reads with terminal Illumina primer and adaptor sequences using Cutadapt 1.1^63^ with the following parameters: times 3, overlap 10, minimum length 25. Reads with a discarded pair <25 bp were kept as singleton reads.

The pre-processed high-quality paired reads of high-alkaloid accession P27255 were assembled *de novo* using four methods: Trinity 2.2.0 with glue and k-mer coverage thresholds of 5^64^; SOAPdenovo-TRANS 1.03 with k-mer length values from 31–71 in steps of 10^65^; VELVET 1.2.10 (with k-mer values from 25–65 in steps of 10) and OASES 0.2.09^66,67^; and CLC Genomics Workbench 9.0.1 with default settings (https://www.qiagenbioinformatics.com/). Because of memory constraints for the VELVET + OASES assembly, the data were further filtered (digitally normalized) with KHMER 2.0^68^ at a uniform coverage value C of 100. Non-redundant transcripts from the four P27255 assemblies were merged using the EvidentialGene tr2aacds pipeline (http://arthropods.eugenes.org/genes2/about/EvidentialGene_trassembly_pipe.html). We chose P27255 as the reference transcriptome based on the assumption that genes involved in QA biosynthesis will be upregulated in the high-alkaloid accessions.

The individual P27255 assemblies were compared based on single-copy ortholog completeness by BUSCO v3^69^ with the Embryophyta reference core genes (dataset embryophyta_odb9). The ortholog completeness assessment results for the currently available Tanjil transcriptome^32^ and the draft NLL genome^34^ were included for comparison. The assembled transcripts were functionally annotated using Blast2GO Pro v.4.1.5 by aligning them against UniProt/SwissProt^70^. The merged P27255 transcriptome sequence has been deposited in GenBank as a transcriptome shotgun assembly (TSA) under accession GGED00000000.

The draft NLL genome^34^ was used as the reference to determine the genome positions of the investigated QA candidates (denominated as TanjilG_number in the NLL genome at NCBI and equivalent to Lup_number of Hane, *et al*.^34^). The precise exon/intron structures of selected QA candidate genes were assessed based on the predicted protein sequence of the transcript and the draft NLL genome using WebScipio^71^.

### Differential gene expression and selection of QA candidate genes

Differentially expressed genes between bitter and sweet NLL accessions were detected using RSEM 1.2.28^72^. Twelve candidate genes were selected for thorough investigation (Table 2) using two different approaches:

#### Analysis of the assembled P27255 transcriptomes

As sweet NLL accessions are known to produce low amount of alkaloids we assumed that they possess genetic determinants required for biosynthesis. Therefore candidate DEGs that were overexpressed in the bitter accessions were filtered based on multiple serially applied criteria. First, only DEGs with a maximum posterior probability of equal expression value of 0.0001 (posterior probability that a gene/transcript is equally expressed ≤0.0001, which corresponds to having used the rsem-control-fdr algorithm with default hard threshold parameter turned on) were selected. Second, from among these DEGs, only those with more than two-fold higher expression than the sweet accessions baseline were selected (RSEM was used to estimate fold changes between groups; PostFC >2, see Supplementary Table S1 for full list of candidate genes.) Third, the selected DEGs were further filtered to retain only those with read counts in both the sweet and bitter accessions. Finally, based on the annotations and GO terms, 10 candidate genes related to plant secondary metabolism and its regulation were selected for an in-depth investigation. QA biosynthesis pathway is not well understood regarding candidate enzymes involved in oxidation/reduction processes leading to ring closure^4,5,7–9^ as well as modifications of the final QA products. Therefore we also considered singular representatives for the oxidoreductive enzyme families and transcription factors known to be involved in other secondary metabolism pathways, both due to possible involvement in QA modification and due to possibility of correlation/cross-talk between different metabolite groups. Additionally, a candidate locus annotated as *HMT/HLT* was selected for further analyses despite its equal expression in the bitter and sweet accessions RNA-Seq libraries, because of its verified role in QA biosynthesis^11^.

#### Analysis of the iucundus region in pseudochromosome NLL-07

We assumed that some candidate DEGs may be present in the draft NLL genome, particularly in the region of the major alkaloid *iucundus* locus in pseudochromosome NLL-07 (i.e., between markers LaDArT_PAV21233 and LaSSR_025 on the molecular genetic map of Hane, *et al*.^34^ corresponding to physical interval NLL-07:14591111–15337202). Two DEGs were found in this region: P27255_011214 (equivalent to TanjilG_017658 by reciprocal BLAST search) predicted to encode 4-hydroxy-tetrahydrodipicolinate synthase (DHDPS), and P27255_002404 (equivalent to TanjilG_007701) annotated as metalloprotease and, hence unlikely to be associated with QA biosynthesis. We selected only P27255_011214 for further analyses.

### Linkage mapping of candidate QA genes

PCR primers were designed using both the assembled transcripts and pre-processed raw read sequences. Transcriptome-derived SNP markers were designed for the 12 selected candidate QA genes (Table 2). The primer sequences and PCR thermal conditions are listed in Supplementary Table S3. Segregation analysis was performed for 93 RILs. Transcriptome-derived markers were distributed within the linkage groups of the reference genetic map^34^ limited to framework markers and six trait loci (total 2963 loci) using MapManager QTXb20^73^. The initial map imported to MapManager software (based on the 2963 loci) was 2438.2 cM, with average interval size 0.82 cM (compared with 2500.8 cM and 0.85 cM, respectively, in the genetic map of Hane, *et al*.^34^). For each locus the observed segregations were tested against the expected Mendelian segregation ratio (1:1) using the chi-square test. The LGs was visualized using MapChart^74^.

### Validation of gene expression by qRT-PCR

To confirm the expression levels obtained using the RNA-Seq data, we performed qRT-PCRs for 12 QA candidates, including 11 DEGs and one gene (*HMT/HLT*) that was expressed constitutively in the sweet and bitter accessions. First, seven housekeeping genes in the NLL transcriptome, namely actin 2/7 (*ACT2/7*), *ADH3*, *ATPsyn*, cyclophilin (*CYP*), elongation factor 1-beta (*ELF1B*), glucose-6-phosphate 1-dehydrogenase (*G6PD*), *TUBA* were tested as candidate reference genes for the qRT-PCRs. We selected the three most stable reference genes, *TUBA*, *ATPsyn*, and *ADH3*, to normalize the expression levels of the selected QA candidate genes (see Supplementary Data S1 for details).

Seven low-alkaloid and seven high-alkaloid NLL accessions were selected for the qRT-PCR analyses (Table 1). Primer pairs and probes were designed based on DNA sequence of candidate QA and reference genes for the 83A:476 and P27255 accessions. The qRT-PCRs were performed as one-step reactions using LightCycler 480 RNA Master Hydrolysis Probes on a LightCycler480 system (Roche, Mannheim, Germany) according to the manufacturer’s protocol (reaction volume 10 µl). Three biological and two technical replicates of each accession, as well as a negative control, were included in each assay. PCR amplification efficiencies ranged from 0.97 to 1.09 (Supplementary Table S8), as determined using a standard curve derived from a pooled RNA mixture. The primer/probe sequences and reaction conditions for the qRT-PCRs are listed in Supplementary Table S8.

Gene expression was analysed using the delta-delta Ct method with the three reference genes (*TUBA*, *ATPsyn*, and *ADH3*). All Cq values were subjected to efficiency corrections^75^. Statistical testing of differences in mean Cq values was conducted using the Wilcoxon signed rank test corrected for multiple comparisons (Bonferroni correction; final P-value threshold of 0.0001).

### QTL mapping and statistical analysis of the bitter/sweet phenotypes

Phenotypic data distribution was evaluated across 5 years for both total QA content and relative abundance of four major QAs. Variance components for years and lines were estimated by the restricted maximum likelihood method, and broad-sense heritability was computed using the formula of Cullis, *et al*.^76^. A principal component biplot was created to visualize the distribution of of total QA content and relative abundance of individual QAs in the sweet and bitter subgroups of RILs established based on their *iucundus* allele. These analyses were performed in Genstat 18^77^.

QTL analysis was conducted on mean values for RILs, for each year separately using the composite interval mapping approach in the QTL Cartographer 2.5 software^78^. The genome was scanned at a walking speed of 1 cM. Significance threshold values of LOD for QTL detection were determined by a permutation test with 1000 replicates (P value < 0.05). The percentage variations explained by each QTL and additive effect were estimated. The QTL confidence intervals were determined as one-LOD and two-LOD support intervals^79^.

### Phylogeny of ethylene-responsive TF (*RAP2-7*) homologs in Viridiplantae genomes

Available Viridiplantae genomes (Supplementary Table S9) were annotated with known *ERF*s using iTAK software based on the presence of sequence signatures of associated protein domains and conserved motifs^80^. The identified AP2/ERF TFs were compared all-against-all (USEARCH/UBLAST) and clustered using the Markov cluster algorithm^81^ based on expectation values. Clustering quality was tested based on silhouette width as described by Koczyk, *et al*.^82^. The optimal parameter to discriminate the cluster containing candidate NLL TFs was set as I = 1.7. For phylogeny reconstruction, all sequences <200 amino acids in length were removed from the cluster, the remaining sequences were subsequently aligned using MAFFT-LINSI^83^. In particular, several partial sequences derived from *Selaginella*, *Coccomyxa*, and *Chlamydomonas*, as well as one *Medicago truncatula* gene sequence (Mtr_7g061190) were rejected as too short and/or lacking class-specific conserved residues. Finally, a maximum-likelihood tree was constructed by IQTREE 1.5.5 based on aligned protein sequences^84^ (ultrafast bootstrap replicates threshold set to 3000). The evolutionary model was selected by the program according to the Bayesian information criterion (JTT + F + I + G4). *Chlamydomonas reinhardtii* and *Volvox carteri* sequences represented the outgroup that was used to root the final tree.

## Supplementary information

Supplementary Table S1.

Supplementary Table S2.

Supplementary Tables S3-S9

Supplementary Figures S1-S2.

Supplementary Data S1.

## Data Availability

Transcriptome Shotgun Assembly project has been deposited in GenBank under the accession GGED00000000. RNA-Seq data have been submitted as short-read archives under the following accessions: SRR5723679, SRR5723680, SRR5723681, and SRR5723682 in BioProject PRJNA389154.
