# Supplementary material for: Transcriptome-derived investigation of biosynthesis of quinolizidine alkaloids in narrow-leafed lupin (Lupinus angustifolius L.) highlights candidate genes linked to iucundus locus
Source: Sci Rep. 2019 Feb 19;9:2231. doi: 10.1038/s41598-018-37701-5 (PMC6381137; doi:10.1038/s41598-018-37701-5)
Supplement: Supplementary file 3 — Supplementary Tables S3-S9 [file 41598_2018_37701_MOESM3_ESM.pdf]

"Transcriptome-derived investigation of biosynthesis of quinolizidine alkaloids in narrow-leafed lupin (*Lupinus angustifolius* L.) highlights candidate genes linked to *iucundus* locus".

Magdalena Kroc, Grzegorz Koczyk, Katarzyna A. Kamel, Katrzyna Czepiel, Olga Fedorowicz-Strońska, Paweł Krajewski, Joanna Kosińska, Jan Podkowiński, Paulina Wilczura and Wojciech Święcicki

Supplementary Tables S3-S9

Supplementary Table S3. PCR primers and reaction conditions, and linkage mapping of candidate alkaloid genes

**Marker symbol**	**PCR**		**PCR thermal profile (°C)**	**Polymorphism detection method /restriction enzyme**	**Linkage group (NLL)**	**LOD**	**χ2**
	**Forward primer seq**	**Reverse primer seq**					
CCR	CAAATCCGATATCCCCAACA	AGCCTCTCCTTTGCTCCTTC	54	CAPS/TasI	NLL-02	26.5	0.2
DFR1	GAATGGGTTCTACACATATTG	AACAGGTTTCGTCATAGTCA	50	CAPS/TspRI	NLL-03	27.1	0.6
HMT/HLT[†]/[‡]	TGTTTGGGATACCAACAAATG	CGACTACTGAAATGTGAGGAGAAA	60	CAPS/HinfI	NLL-04	19.4	0.9
CES1L	GCCTCCTCACAATCTCAA	TATCTGCCATGTCCACACAGAAATCATGGC	55	dCAPS/BsuRI	NLL-08	28.0	0.5
LDC[†]	TATGCGAACCCATGCAAAT	GCTGTTGTGTTAAACCCATTGA	56	CAPS/StyI	NLL-15	27.4	1.1
LaCAO[†]	TATATTCTGTTGCATATGTTGATGGTAGTCAAGC	TTTGACTATGCCAGAACAATCACATC	55	dCAPS/HhaI	NLL-15	20.2	1.1
LaAT[†]	CGTGTCAATATATGATTAGTTTGAGACTGA	TTGCTTTCACTGGTGATTTTATC	50	dCAPS/DdeI	NLL-16	24.4	0.2
F3H	GCTCCCTGAATAGGTTGAACAGTAATCCAAAT	CAAACAAGCCAGAAGGGTGGATAAAAGTGA	55	dCAPS/XapI	NLL-17	27.7	0.5
LDOX	CTGAAACATTCAAGGGTACG	ATAGACAAGTCACGTTTATCTT	50	CAPS/MboI	NLL-18	23.5	1.8
RAP2-7	TCAATCCAAGTCCTTTTGTCC	TGCATAGAAAATTAAACACTTTTTTAAGC	54	dCAPS/AluI	NLL-07	27.4	10.8
MYB	GAAATCAAGAACTACTGGAACT	CAGAGCATTATTATTGGCACT	55	CAPS/VspI	NLL-15	24.1	1.5
DHDPS	TTTCTGTGTGGAGTGGGAAT	ATAAAACCAGGAACCAGGTTGCTTACGGC	50	dCAPS/HaeIII	NLL-07	27.7	8.7

[†]Known quinolizidine alkaloid biosynthesis genes that have already been described for lupins.
[‡]This gene had similar expression levels in both sweet and bitter NLL accessions.

Supplementary Table S4. Mean values of total quinolizidine alkaloid (QA) content and relative abundance of individual alkaloids for the RIL subgroups based on the *iucundus* allele (*iucundus* - sweet and *Iucundus* - bitter).

Iucundus allele	Total QA content	Relative abundance			
		Lupanine	13-hydroxylupanine	Angustifoline	Isolupanine
iucundus = sweet	0.545	68.8	18.26	8.95	4.309
Iucundus= bitter	1.792	68.4	17.75	11.78	2.125

Supplementary Table S5. Normality test for total quinolizidine alkaloid (QA) content and relative abundance of individual alkaloids. „*” means normality rejected at P < 0.01.

**Variate**	**Year**	**Cramer-von Mises statistic**	**Normality rejected at P<0.01**
Total QA content	2008	0.41	*
	2010	0.27	*
	2011	0.34	*
	2013	0.66	*
	2015	0.56	*
Lupanine	2008	0.09	
	2010	0.19	*
	2011	0.07	
	2013	0.18	*
	2015	0.12	
13-hydroxylupanine	2008	0.07	
	2010	0.14	
	2011	0.04	
	2013	0.13	
	2015	0.09	
Angustifoline	2008	0.42	*
	2010	0.21	*
	2011	0.11	
	2013	0.07	
	2015	0.09	
Isolupanine	2008	0.77	*
	2010	0.66	*
	2011	0.31	*
	2013	1.02	*
	2015	0.69	*

Supplementary Table S6. Assessment of variance components for years and lines (with standard errors) and broad sense heritability for total quinolizidine alkaloid (QA) content and relative abundance of individual alkaloids.

Trait	Year		Lines		Heritability (%)
	Variance	s.e.	Variance	s.e.	
Total QA content	0.14	0.5	0.01	0	91.15
Lupanine	18.39	14	175.15	9.3	72.82
13-hydroxylupanine	7.33	4.5	27.84	1.1	67.86
Angustifoline	2.67	4.9	3.79	1	79.84
Isolupanine	0.24	8.8	0.04	2.2	93.96

Supplementary Table S7. Map locations of the QTLs for total quinolizidine alkaloid (QA) content and relative abundance of individual alkaloids in the draft NLL genome across five years. Stable QTLs have been highlighted.

QTL symbol	Linkage group (NLL)	Closest marker	Position (cM)	LOD	a[†]	R2 (%)[‡]	1-LOD support interval (cM)	2-LOD support interval (cM)
Total_QA_2008	NLL-07	*Iucundus*/RAP2-7	78.7	27.8	-0.68	70.7	77.7 - 83.5	77.2 - 88.3
Total_QA_2010	NLL-06	LaDArT_PAV14830	68.2	4.5	-0.41	10.8	67.2 - 69.8	63.9 - 71
Total_QA_2010	NLL-07	LaDArT_SNP07619	82.1	7.8	-0.55	18.9	80.5 - 83.2	79.3 - 83.7
Total_QA_2010	NLL-16	LaSNP_040 /LaDArT_SNP11122	1.7	3.8	0.35	8.9	0 - 5.1	0 - 7.4
Total_QA_2011	NLL-07	*Iucundus*/RAP2-7	78.7	16.7	-0.77	48.1	77.6 - 86.8	77 - 95
Total_QA_2013	NLL-07	LaSNP_475	81	16.6	-0.57	49.1	80.1 - 82.5	79.7 - 83.3
Total_QA_2013	NLL-10	LaDArT_SNP14644	72.5	4.9	-0.23	8.9	71.2 - 75.3	68.4 - 76.5
Total_QA_2013	NLL-20	LaDArT_PAV15790	82.9	5.8	0.25	10.7	81 - 83.9	79.6 - 84.8
Total_QA_2015	NLL-07	*Iucundus*/RAP2-7	78.7	16.2	-0.42	44.5	77.7 - 79.2	78.4 - 79.3
Lupanine_2008	NLL-04	LaDArT_SNP18757	119.7	2.8	3.92	7.4	115.6 - 121.4	112.9 - 121.4
Lupanine_2010	NLL-01	LaDArT_SNP03034	26.4	1.9	2.88	7.3	17.8 - 33.6	26.4 - 26.4
Lupanine_2010	NLL-06	LaDArT_SNP11608	6.7	3.3	-4.05	12.7	0 - 8.4	0 - 17.6
Lupanine_2011	NLL-01	LaDArT_SNP03362	87.4	3.8	-2.43	12.6	86.2 - 87.9	85.7 - 87.9
Lupanine_2011	NLL-20	LaDArT_SNP10376	57	3.9	-2.36	13.4	54.8 - 58.3	54.8 - 58.6
Lupanine_2013	NLL-10	LaDArT_SNP06483	56	2.8	3.31	9.5	54 - 57.1	52 - 57.1
Lupanine_2013	NLL-19	LaDArT_SNP04488	3.5	3.4	-3.67	11.7	0.3 - 10.2	0 - 11
Lupanine_2015	NLL-08	LaDArT_SNP13113	149.4	2.6	3.28	10.2	147.7 - 149.9	146.5 - 149.9
13-hydroxylupanine_2008	NLL-02	LaDArT_SNP06159	95.5	6.1	2.13	19.3	92 - 96.5	90.9 - 100.7
13-hydroxylupanine_2008	NLL-03	LaDArT_SNP00899	117.7	5.7	2.19	18.3	114.1 - 120.8	110.9 - 123.1
13-hydroxylupanine_2008	NLL-04	LaDArT_SNP07454	29.3	3.4	1.55	10.0	27.6 - 30.5	26.8 - 30.5
13-hydroxylupanine_2010	NLL-06	lPb-331382	87.5	5.9	1.82	22.8	85.5 - 90.3	85 - 93.3
13-hydroxylupanine_2011	NLL-01	LaDArT_SNP04894	92.4	4.4	1.68	13.9	91 - 93.4	90.2 - 93.6
13-hydroxylupanine_2011	NLL-05	LaDArT_SNP03039	22.1	5.3	-1.87	17.9	20.4 - 23.3	20.4 - 25
13-hydroxylupanine_2013	NLL-04	LaDArT_SNP17267	127.2	3.4	-1.76	11.8	122.5 - 130.2	122.5 - 133.9
13-hydroxylupanine_2013	NLL-13	LaDArT_PAV22279	69.7	3.9	1.85	13.3	69.1 - 71.1	69.1 - 74.6
13-hydroxylupanine_2015	NLL-06	LaDArT_PAV17258	23.3	3.9	2.09	14.0	17.2 - 23.9	12.7 - 23.9
13-hydroxylupanine_2015	NLL-15	LaDArT_SNP05410	58.9	3.4	1.99	12.0	56.1 - 61.1	54.4 - 64.5
Angustifoline_2008	NLL-07	*Iucundus*/RAP2-7	78.3	21.7	-3.27	56.4	78.1 - 79	77.8 - 79.2
Angustifoline_2008	NLL-08	LaDArT_SNP01123	27.3	5.6	-1.19	8.5	22.6 - 31	19 - 34.6
Angustifoline_2010	NLL-07	LaDArT_SNP07310	63.2	3.9	-1.02	14.1	61.5 - 66.2	61.5 - 69.5
Angustifoline_2011	NLL-07	LaDArT_PAV20288	89.6	3.9	-1.40	12.6	89 - 90.8	87.3 - 90.8

Angustifoline_2011	NLL-09	LaDArT_PAV22879	116	4.8	1.61	16.7	111.9 - 117	110.7 - 117.7
Angustifoline_2013	NLL-10	LaDArT_SNP06483	56	7.8	-2.38	27.0	55.4 - 60.4	55.4 - 56.6
Angustifoline_2013	NLL-13	LaDArT_SNP06999	35.1	7.2	1.96	24.0	34.3 - 36.1	33.4 - 36.8
Angustifoline_2015	NLL-07	*Iucundus*/RAP2-7	78.7	5.6	-1.64	18.1	76.4 - 80.8	74.5 - 82.8
Isolupanine_2008	NLL-07	*Iucundus*/RAP2-7	78.7	4.6	1.33	13.7	78.7 - 80.6	77.5 - 83.9
Isolupanine_2008	NLL-08	LaDArT_PAV10464	85.2	9.9	-1.90	34.2	83.6 - 86.4	83.6 - 88.3
Isolupanine_2008	NLL-10	LaDArT_PAV24457	89.7	2.6	0.88	7.1	80.1 - 90.7	77.5 - 90.7
Isolupanine_2010	NLL-07	*Iucundus*/RAP2-7	78.7	3.6	1.19	11.4	77.8 - 81.4	76.3 - 83.5
Isolupanine_2010	NLL-08	LaDArT_SNP06932 /CES1L	84.7	7.2	-1.59	25.2	83.7 - 86.5	83.6 - 88.4
Isolupanine_2011	NLL-08	LaDArT_SNP06932 /CES1L	84.7	8.3	-1.55	25.3	84.5 - 86.5	83.6 - 90.2
Isolupanine_2013	NLL-07	LaSNP_278	80.4	6.9	2.28	20.3	79.3 - 81.8	79.3 - 83.1
Isolupanine_2013	NLL-08	LaDArT_SNP03779	84.1	10.0	-2.33	31.7	83.6 - 84.9	83.6 - 86.5
Isolupanine_2013	NLL-14	LaDArT_SNP11318	85.3	2.6	1.12	7.2	83.1 - 85.8	81.7 - 85.8
Isolupanine_2015	NLL-05	LaDArT_SNP02227	15.7	2.7	0.89	7.1	10.8 - 21.7	8.8 - 27.1
Isolupanine_2015	NLL-07	LaSNP_475	81	6.4	1.46	18.9	79.3 - 83.3	79.3 - 84.7
Isolupanine_2015	NLL-08	LaDArT_SNP12470	80.1	9.7	-1.80	31.5	79.6 - 81.3	79 - 81.3

† Additive effect

‡ The amount of phenotypic variation explained by a QTL (%)

Supplementary Table S8. Primer/probe sequences and reaction conditions for the qRT-PCRs of candidate alkaloid genes

Gene /Marker symbol	P27255 transcript name	PCR thermal profile (°C)	Final concentration in reaction (μl) †			Primer sequence	Probe sequence	Product length (bp)	Efficiency
			Primer pairs	Probes	Template RNA				
CCR	P27255_010872	59	0.6	0.25	25	F: ACCGGAGCTGGTGGATTTAT<br>R: TGGGTTCCTTAGGGTTCCTC	TGGATTGTCAAACTTCTCTTACACAAAGGC	87	0.97
CES1L	P27255_012169	58	0.4	0.1	25	F: CCACCCACTTCAGATCCCAA<br>R: CCATGACTTGATAATAATGTTCTTC	CCCTGTCCTCACCAAAGATGTCACCATTA	122	0.97
DFR1	P27255_011759	58	0.3	0.1	25	F: AGGCACATTATTCAATCATTAAGCA<br>R: CCTTCCTTCTACTTTTGGCAG	CTTAGATGATCTTTGTCTTGCTCACATATTCA	101	0.94
F3H	P27255_010739	60	0.5	0.1	25	F: TTTCATTGTCAACCTTGGTGATC<br>R: ACGGCTACAGTTAGAGTTCACTA	ATTATCTGAGCAATGGAAGGTTCAAGAATGC	97	1.03
HMT /HLT	P27255_007723	58	0.4	0.25	25	F: ATTACAACCCGAAAACCCTAATCA<br>R: GCTGCTGGTGACACAAATGC	CAACCCTCCACTCCCTGAAGGCTTTTA	129	0.99
LaAT	P27255_007730	56	0.5	0.2	25	F: CCACCTTCCAAAGCCTTATT<br>R: GTGTTGCCATGCCTAAGTTT	TAACCTTCAGAGCTTCCATCTCCTCTC	146	1.01
LaCAO	P27255_002184	60	0.5	0.1	25	F: TCCTAATCAAAATCCACGCATTG<br>R: GTGACTCCAAATACATACCAAAG	CCTACATGGGTTACGCAGAACCGATCT	105	0.98
LDC	P27255_008143	58	0.4	0.25	25	F: ATTGGTGGCGGTTTCACTTG<br>R: AAAGGTGACTCAGCAAAATAACG	TGGAAAAGAGGAAGGTGTTGTGGTAATTGGA	140	1.09
LDOX	27255_009671	58	0.4	0.1	25	F: CAGTGGAATACAATCAATACCC<br>R: TCTTTCAAGTCAATGGTTGGAAC	CCAGAAGAAGAGTTGAGTAACATAGCCAAT	126	0.98
MYB	P27255_010054	58	1	0.1	25	F: AAGGTGTTGATCCATTTAGTACC<br>R: AGATGACTCTGATTTTGATGAGC	TTACCACGGTTAGTTCTTCATCAGATTCAG	101	0.97
RAP	P27255_008724	58	0.8	0.2	25	F: TATCACATTCTGGGGCATCC<br>R: CCACTCCCTTGTTCCTTTCA	TCCTGTGGAGGAAGATTCCTCAAACAA	135	1.07
DHDPS	P27255_011214	60	0.5	0.1	25	F: TGTGTGGAGTGGGAATGATGA<br>R: TCAGAGTAGGGTTGTTGCCG	ATGATGCTAGATGGGGTTTCGGGGCTA	137	1.03

† Total reaction volume 10 (μl)

Supplementary Table S9. Viridiplantae genomes used in the phylogenetic analysis

No.	**Genome**
1	*Coccomyxa subellipsoidea*
2	*Chlamydomonas reinhardtii*
3	*Volvox carteri*
4	*Physcomitrella patens*
5	*Selaginella moellendorffii*
6	*Spirodela polyrhiza*
7	*Zostera marina*
8	*Musa acuminata*
9	*Ananas comosus*
10	*Setaria italica*
11	*Sorghum bicolor*
12	*Zea mays*
13	*Brachypodium distachyon*
14	*Hordeum vulgare*
15	*Oryza sativa*
16	*Beta vulgaris*
17	*Amaranthus hypochondriacus*
18	*Vitis vinifera*
19	*Mimulus guttatus*
20	*Solanum lycopersicum*
21	*Solanum tuberosum*
22	*Fragaria vesca*
23	*Malus domestica*
24	*Prunus persica*
25	*Cucumis sativus*
26	*Arachis ipaensis*
27	*Lupinus angustifolius*
28	*Lotus japonicus*
29	*Medicago truncatula*
30	*Trifolium pratense*
31	*Cajanus cajan*
32	*Glycine max*
33	*Phaseolus vulgaris*
34	*Vigna radiata*
35	*Ricinus communis*
36	*Populus trichocarpa*
37	*Salix purpurea*
38	*Citrus sinensis*
39	*Theobroma cacao*
40	*Carica papaya*
41	*Brassica rapa*
42	*Capsella grandiflora*
43	*Arabidopsis thaliana*
