# Supplementary material for: Transcriptome-derived investigation of biosynthesis of quinolizidine alkaloids in narrow-leafed lupin (Lupinus angustifolius L.) highlights candidate genes linked to iucundus locus
Source: Sci Rep. 2019 Feb 19;9:2231. doi: 10.1038/s41598-018-37701-5 (PMC6381137; doi:10.1038/s41598-018-37701-5)
Supplement: Supplementary file 4 — Supplementary Figures S1-S2. [file 41598_2018_37701_MOESM4_ESM.pdf]

"Transcriptome-derived investigation of biosynthesis of quinolizidine alkaloids in narrow-leafed lupin (*Lupinus angustifolius* L.) highlights candidate genes linked to *iucundus* locus".

Magdalena Kroc, Grzegorz Koczyk, Katarzyna A. Kamel, Katrzyna Czepiel, Olga Fedorowicz-Strońska, Paweł Krajewski, Joanna Kosińska, Jan Podkowiński, Paulina Wilczura and Wojciech Święcicki

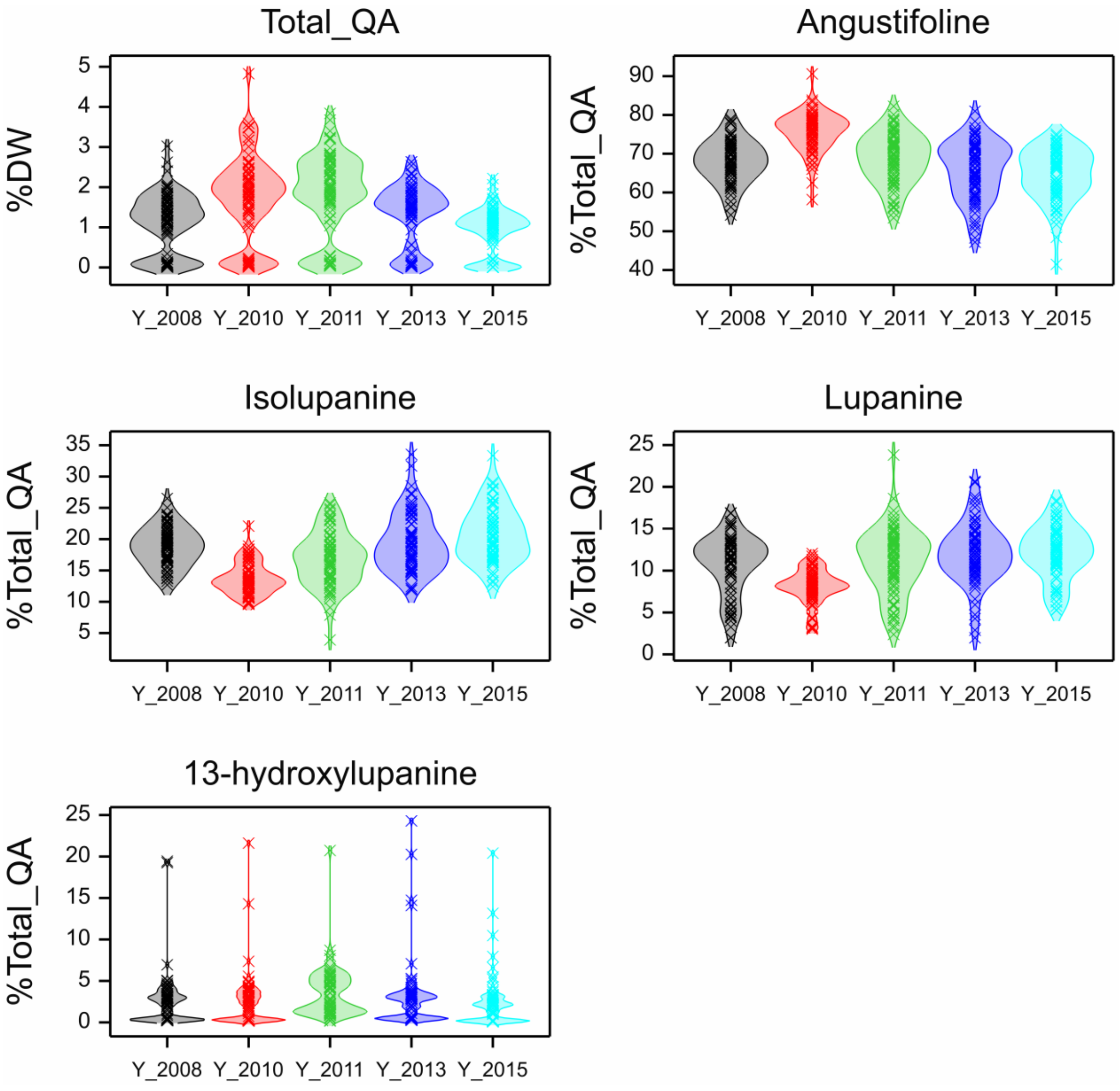

Supplementary Figure S1. Distribution of total quinolizidine alkaloid content and relative abundance of individual alkaloids across years.

"Transcriptome-derived investigation of biosynthesis of quinolizidine alkaloids in narrow-leafed lupin (*Lupinus angustifolius* L.) highlights candidate genes linked to *iucundus* locus".

Magdalena Kroc, Grzegorz Koczyk, Katarzyna A. Kamel, Katrzyna Czepiel, Olga Fedorowicz-Strońska, Paweł Krajewski, Joanna Kosińska, Jan Podkowiński, Paulina Wilczura and Wojciech Święcicki

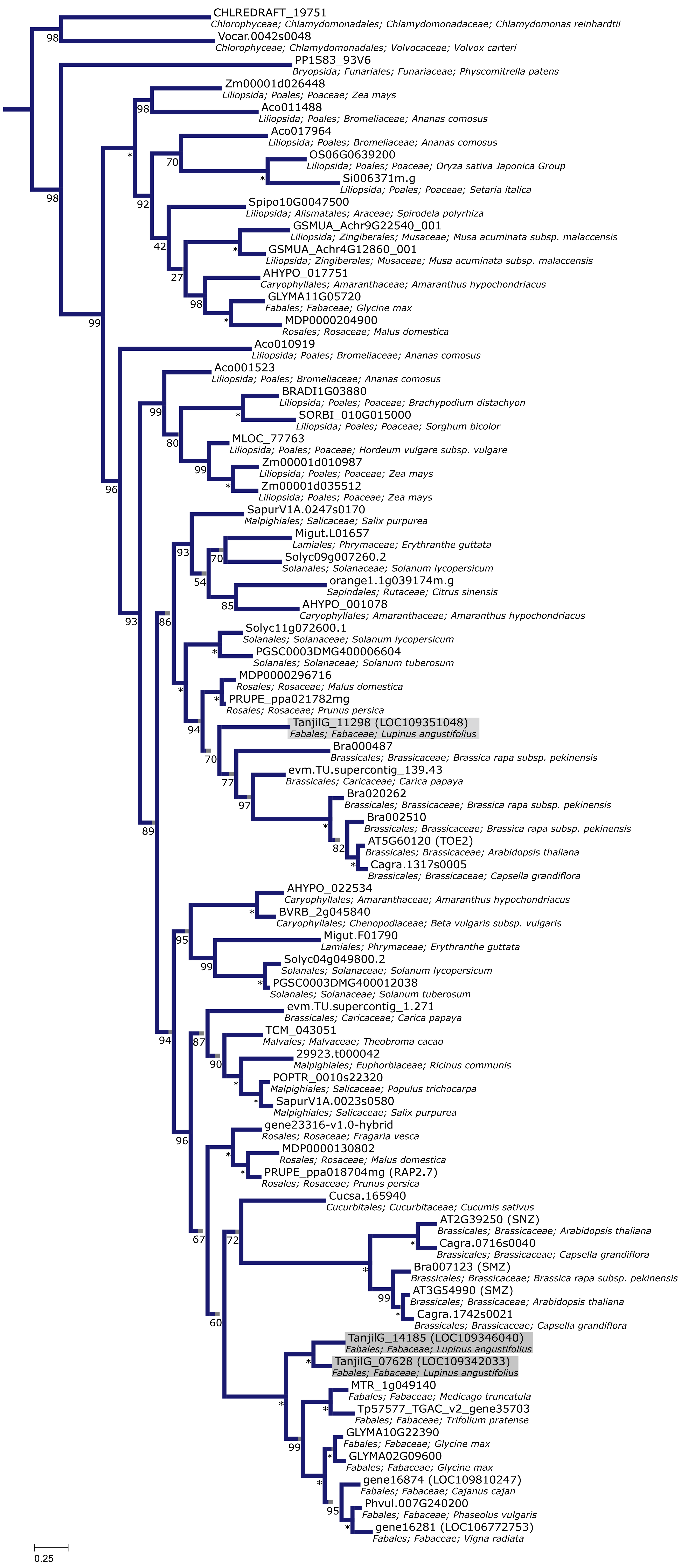

Supplementary Figure S2. Maximum-likelihood phylogenetic tree of the candidate AP2/ERF TF and related genes in Viridiplantae genomes
