# Supplementary material for: Transcriptome-derived investigation of biosynthesis of quinolizidine alkaloids in narrow-leafed lupin (Lupinus angustifolius L.) highlights candidate genes linked to iucundus locus
Source: Sci Rep. 2019 Feb 19;9:2231. doi: 10.1038/s41598-018-37701-5 (PMC6381137; doi:10.1038/s41598-018-37701-5)
Supplement: Supplementary file 5 — Supplementary Data S1. [file 41598_2018_37701_MOESM5_ESM.pdf]

"Transcriptome-derived investigation of biosynthesis of quinolizidine alkaloids in narrow-leafed lupin (*Lupinus angustifolius* L.) highlights candidate genes linked to *iucundus* locus".

Magdalena Kroc, Grzegorz Koczyk, Katarzyna A. Kamel, Katrzyna Czepiel, Olga Fedorowicz-Strońska, Paweł Krajewski, Joanna Kosińska, Jan Podkowiński, Paulina Wilczura and Wojciech Święcicki

Supplementary Data S1

Evaluation and selection of candidate reference genes for qRT-PCR studies in narrow-leafed lupin.

Aim: To evaluate and select appropriate reference genes for qRT-PCRs to analyze the expression levels of quinolizidine alkaloid (QA) genes in narrow-leafed lupin (NLL).

Method: We selected seven housekeeping genes as candidate reference genes (actin 2/7 (*ACT2/7*), alcohol dehydrogenase class-3 (*ADH3*), ATP synthase (*ATPsyn*), cyclophilin (*CYP*), elongation factor 1-beta (*ELF1B*), glucose-6-phosphate 1-dehydrogenase (*G6PD*), and alpha tubulin (*TUBA*)) (Table A1). The primer/probe sequences and PCR thermal conditions are listed in Table A2. The qRT-PCRs were performed as one-step reactions using LightCycler 480 RNA Master Hydrolysis Probes on a LightCycler480 system (Roche, Mannheim, Germany) according to the manufacturer's protocol (reaction volume 10 µl), on seven low-alkaloid and seven high-alkaloid NLL accessions (Table 1 in the original paper). Three biological and two technical replicates of each accession, as well as a negative control, were included in each assay. Then, we used geNorm, NormFinder, Bestkeeper, and the delta Ct method implemented in the web-based RefFinder[1] software to analyze their expression stabilities. GenEx Std v. 6.0.5.225 software (MultiD Analyses AB, Goteborg, Sweden) was used to determine the optimal number of reference genes by calculating the accumulated standard deviation, as well as to validate the RefFinder results (because RefFinder also incorporates geNorm and NormFinder software). qRT-PCR amplification efficiencies of the candidate reference genes ranged from 0.97 to 1.13 (Table A2), as determined using a standard curve derived from a pooled RNA mixture. To rank the most suitable reference genes, the arithmetic mean of the Ct values for the two technical repeats of each biological replicates was imported to both RefFinder and GenEx.

Results and discussion: Using two different approaches (RefFinder and GenEx), we identified not only genes with the most stable expression patterns but also selected the

minimum number of internal control genes. While the rank order built by individual statistical approaches was not identical, the most and least stable reference genes were consistent among all analyses (Table A3). A minimum was achieved in the accumulated standard deviation plot (0.3), as assessed by GenEx, when the three top-ranked reference genes were used (Figure A1). Therefore, we selected *TUBA*, *ATPsyn* and *ADH3* as the three most suitable reference genes for QA gene expression normalization. A different set of stable reference genes was reported for NLL[2], but the seven genes that we investigated were not evaluated in the previous report. Taylor and coworkers evaluated three promising reference genes in parental NLL lines 83A:476 (low-alkaloid) and P27255 (high-alkaloid) under vernalization treatment, organ type, and developmental stages, and conducted their expression analyses on cDNA, whereas we performed the RT-qPCRs as one-step reactions. It is well known that the stability of basic metabolism genes may be influenced by numerous environmental[3] .

Conclusion: Our evaluation of seven housekeeping genes has broadened the choices available for best normalizing the qRT-PCR results in NLL with respect to increased stability and repeatability in experimental designs focused on QA biosynthesis, and is an important contribution of this study.

1 Xie, F., Xiao, P., Chen, D., Xu, L. & Zhang, B. miRDeepFinder: a miRNA analysis tool for deep sequencing of plant small RNAs. *Plant Mol. Biol.* **31**, 31 (2012).

2 Taylor, C. M., Jost, R., Erskine, W. & Nelson, M. N. Identifying stable reference genes for qRT-PCR normalisation in gene expression studies of narrow-leafed lupin (*Lupinus angustifolius* L.). *PLoS One* **11**, e0148300 (2016).

3 Huggett, J., Dheda, K., Bustin, S. & Zumla, A. Real-time RT-PCR normalisation; strategies and considerations. *Genes Immun.* **6**, 279-284 (2005).

Table A1. Candidate reference genes evaluated in this study.

Gene name	Marker symbol	Description	P27255 transcript name
Actin 2/7	*ACT2/7*	Cytoskeletal protein	P27255_065907
Alcohol dehydrogenase class-3	*ADH3*	Conversion of alcohols and aldehydes or ketones	P27255_031091
ATP synthase	*ATPsyn*	ATP synthesis	P27255_015570
Cyclophilin	*CYP*	Protein folding	P27255_019389
Elongation factor 1-beta	*ELF1B*	Translational elongation	P27255_014770
Glucose-6-phosphate 1-dehydrogenase	*G6PD*	Glucose metabolism	P27255_005810
Alpha tubulin	*TUBA*	Cytoskeletal protein	P27255_007866

Table A2. Primer/probe sequences and reaction conditions for the qRT-PCRs of candidate reference genes

Gene /Marker symbol	P27255 transcript name	PCR thermal profile (°C)	Final concentration in reaction (μl) †			Enhancer	Primer sequence	Probe seq	Product length (bp)	Efficiency
			Primer pairs	Probes	Template RNA					
ACT2/7	P27255_065907	58	0.2	0.1	25	no	F:AGATTTGGCATCACACTTTCTAC<br>R:ATTTGGGTCATCTTCTCTCTGTT	TTCTTACTGAGGCACCCCTTAATCCCAAG	115	1.03
ADH3	P27255_031091	58	0.3	0.1	100	no	F:AGCACACAGCGTAGGCATC<br>R:AGTTGATGAGTACATAACCCACA	TCAAAAGCCTCATTGATCTCTTCAAGAGTC	91	1.11
ATPsyn	P27255_015570	59	0.2	0.2	25	no	F:AGTATGCTGTTCCTGTTCGTCA<br>R:ATGGTGATCTTCTCCTTCTTTAG	TGGAGTTGAAGAACAAATATGAGACAGCAATC	145	0.97
CYP	P27255_019389	58	0.4	0.1	100	no	F:ATCATCCTTCCACCGTGTTATC<br>R:TGCTTCTTTATGAAGTTCTCATCTT	CTTCATGTGCCAGGGAGGTGACTTCA	126	1.03
ELF1B	P27255_014770	58	0.2	0.15	25	no	F:ATGGGTGAATGAATCTGATGGCT<br>R:CCTCTCTGGAAAAACCTATATTT	CAGAAGCATACACTTTTATGTCATCCTTTGTCA	100	1.05
G6PD	P27255_005810	58	0.4	0.1	25	no	F:CCTCCAACCTTGTAGGATGAT<br>R:CGTACCAGAACCGATAACATTC	CTGTCTGAGGTTTTTTAGTTGCTTATACTCAATAA	117	1.13
TUBA	P27255_007866	58	0.2	0.15	25	no	F:CGGGTTAGAAAGTTGGCGGA<br>R:CAACAAGAGAGATCCCAAACC	AGGGTTCTTGGTGTTCAATGCTGTTGGT	102	1.13

Table A3. Candidate reference genes stability values and ranking order based on results from geNorm, NormFinder, BestKeeper and DeltaCt. The genes are ranked from the most stable (1), to the least stable (7).

Ranking	geNorm		NormFinder		BestKeeper		DeltaCt	
	gene	M-value	gene	Stability value	gene	std dev [+/- CP]	gene	Average of std dev
1	*TUBA*	0.71	*TUBA*	0.411	*TUBA*	0.43	*TUBA*	1.61
2	*ATPsyn*		*ELF1B*	0.525	*ADH3*	0.46	*ATPsyn*	1.65
3	*ACT2/7*	0.749	*ATPsyn*	0.538	*ACT2/7*	0.46	*ADH3*	1.67
4	*ADH3*	0.775	*ADH3*	0.782	*ATPsyn*	0.63	*ACT2/7*	1.7
5	*ELF1B*	0.886	*ACT2/7*	0.856	*ELF1B*	0.74	*ELF1B*	1.77
6	*CYP*	0.984	*CYP*	0.971	*CYP*	0.93	*CYP*	1.88
7	*G6PD*	2.236	*G6PD*	5.318	*G6PD*	4.73	*G6PD*	5.36

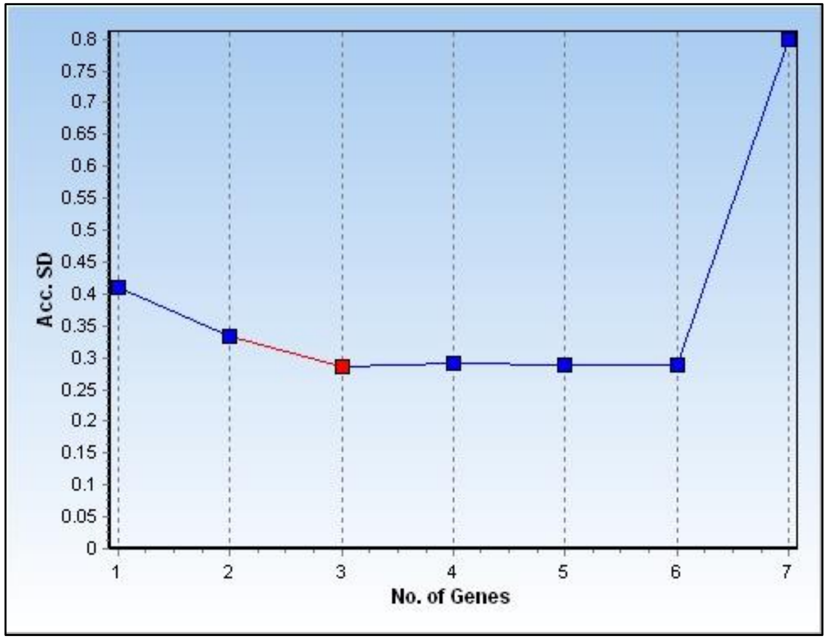

Figure A1. Accumulated standard deviation (Acc. SD) plot to determine the optimal number of reference genes for qRT-PCR normalization
